# Electrically guided versus imaging-guided implant of the left ventricular lead in cardiac resynchronization therapy: a study protocol for a double-blinded randomized controlled clinical trial (ElectroCRT)

**DOI:** 10.1186/s13063-018-2930-y

**Published:** 2018-11-01

**Authors:** Charlotte Stephansen, Anders Sommer, Mads Brix Kronborg, Jesper Møller Jensen, Kirsten Bouchelouche, Jens Cosedis Nielsen

**Affiliations:** 1 Department of Cardiology – Research, Aarhus University Hospital, Palle Juul-Jensens Boulevard 99, DK-8200 Aarhus N, Denmark; 2 Department of Cardiology, Aarhus University Hospital, Palle Juul-Jensens Boulevard 99, DK-8200 Aarhus N, Denmark; 3 Department of Nuclear Medicine, Aarhus University Hospital, Palle Juul-Jensens Boulevard 99, DK-8200 Aarhus N, Denmark

**Keywords:** Heart failure, Cardiac resynchronization therapy, CRT, Left ventricular ejection fraction, LVEF, Electrical mapping, QLV, Cardiac imaging, Left ventricular lead placement

## Abstract

**Background:**

Cardiac resynchronization therapy (CRT) is an established treatment in patients with heart failure and prolonged QRS duration where a biventricular pacemaker is implanted to achieve faster activation and more synchronous contraction of the left ventricle (LV). Despite the convincing effect of CRT, 30–40% of patients do not respond. Among the most important correctable causes of non-response to CRT is non-optimal LV lead position.

**Methods:**

We will enroll 122 patients in this patient-blinded and assessor-blinded, randomized, clinical trial aiming to investigate if implanting the LV lead guided by electrical mapping towards the latest LV activation as compared with imaging-guided implantation, causes an excess increase in left ventricular (LV) ejection fraction (LVEF). The patients are randomly assigned to either the intervention group: preceded by cardiac computed tomography of the cardiac venous anatomy, the LV lead is placed according to the latest LV activation in the coronary sinus (CS) branches identified by systematic electrical mapping of the CS at implantation and post-implant optimization of the interventricular pacing delay; or patients are assigned to the control group: placement of the LV lead guided by cardiac imaging. The LV lead is targeted towards the latest mechanical LV activation as identified by echocardiography and outside myocardial scar as identified by myocardial perfusion (MP) imaging. The primary endpoint is change in LVEF at 6-month follow up (6MFU) as compared with baseline measured by two-dimensional echocardiography. Secondary endpoints include relative percentage reduction in LV end-systolic volume, all-cause mortality, hospitalization for heart failure, and a clinical combined endpoint of response to CRT at 6MFU defined as the patient being alive, not hospitalized for heart failure, and experiencing improvement in NYHA functional class or/and > 10% increase in 6-minute walk test.

**Discussion:**

We assume an absolute increase in LVEF of 12% in the intervention group versus 8% in the control group. If an excess increase in LVEF can be achieved by LV lead implantation guided by electrical mapping, this study supports the conduct of larger trials investigating the impact of this strategy for LV-lead implantation on clinical outcomes in patients treated with CRT.

**Trial registration:**

ClinicalTrials.gov, NCT02346097. Registered on 12 January 2015.

Patients were enrolled between 16 February 2015 and 13 December 2017.

**Electronic supplementary material:**

The online version of this article (10.1186/s13063-018-2930-y) contains supplementary material, which is available to authorized users.

## Background

Cardiac resynchronization therapy (CRT) improves survival, symptoms, and left ventricular (LV) function in patients with medical refractory heart failure and prolonged QRS duration [1–4]. Nevertheless, 30–40% of the patients experience no clinical benefit and the associated risk of complications is non-negligible [5–7].

Potentially correctable causes of non-response to CRT are LV lead positioning and device programming [7, 8]. Guidelines recommend positioning of the LV lead in non-scarred, non-apical, and postero-lateral myocardial segments with late electrical activation [1]. Recent randomized controlled trials have demonstrated improved response to CRT when applying an imaging-guided LV lead placement strategy targeting the latest mechanically activated, non-scarred, myocardial segment, compared with standard care [9–11]. However, imaging-guided strategies are time-consuming, costly, and difficult to align with fluoroscopic imaging during device implantation.

An alternative approach for individualized LV lead placement is to target the myocardial region with the latest electrical activation; a strategy with the advantage of electrical measurements being immediately available during the implant procedure. Retrospective studies have documented an association between pacing the LV in a region with late electrical activation and improved outcome after CRT [12, 13]. However, these studies did not perform systematic electrical activation mapping or evaluate the influence of myocardial scar tissue.

Recently, electrical resynchronization with narrowing of the QRS width during CRT has been associated with better outcome [14]. Furthermore, programming of the interventricular pacing delay (VVd) to achieve the shortest QRS duration is suggested to increase CRT response [15, 16]. Whether the outcome of CRT is improved when combining electrically guided LV lead placement and optimizing the VVd to achieve the shortest QRS duration is unknown.

To clarify the potential value of an electrically guided strategy for optimizing CRT response, we designed a prospective, double-blinded, randomized trial comparing an electrically guided CRT strategy with an imaging-guided approach. We hypothesize that an electrically guided CRT strategy improves CRT response by optimal electrical resynchronization targeting LV lead placement towards the region with the latest electrical activation combined with VVd optimization to achieve the narrowest biventricular paced QRS. The aim of the present study is to investigate if this strategy of optimal electrical resynchronization causes an excess improvement in LV ejection fraction (LVEF) as compared with an imaging-guided strategy positioning the LV lead according to the latest mechanically activated non-scarred myocardial segment.

## Methods

### Study design

The Electrically versus Imaging-guided Implant of the Left Ventricular Lead in Cardiac Resynchronization Therapy Study (ElectroCRT) is a prospective, patient-blinded and assessor-blinded, single-center, randomized, controlled trial. Study participants are allocated 1:1 to either the intervention group or the control group as follows:

1. Intervention group (electrically guided) including pre-implant cardiac computed tomography (CT) to visualize cardiac venous anatomy, procedural electrical activation mapping of all available coronary sinus (CS) branches, placement of the LV lead to pace the site of latest electrical activation, and procedural VVd optimization to achieve the shortest possible QRS duration.
2. Control group (imaging-guided) including pre-implant cardiac CT to visualize cardiac venous anatomy, speckle-tracking echocardiography to identify the LV myocardial segment with the latest mechanical activation, and myocardial perfusion (MP) imaging (^82^Rubidium positron emission tomography (Rb-PET)) to localize the LV myocardial scar. The LV lead is targeted towards the CS branch closest to the latest mechanically activated non-scarred segment and simultaneous biventricular stimulation is applied [17].

**Fig. 1:**
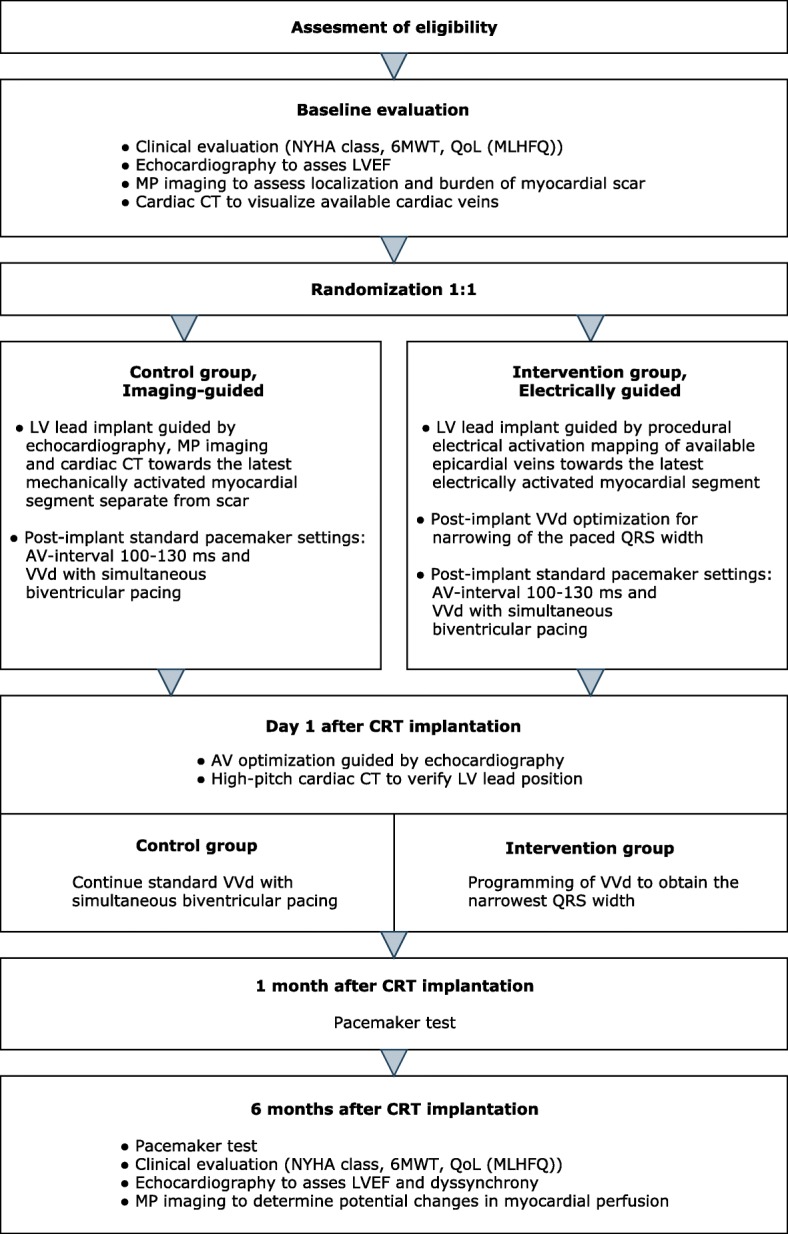
Consort diagram of the study course. 6MWT, 6-minute walk test; AV, atrioventricular; CRT, cardiac resynchronization therapy; CT, computed tomography; LV, left ventricular; LVEF, left ventricular ejection fraction; MLHFQ, Minnesota Living with Heart Failure Questionnaire; MP, myocardial perfusion; NYHA, New York Heart Association; QoL, quality of life; VVd, interventricular pacing delay

**Fig. 2:**
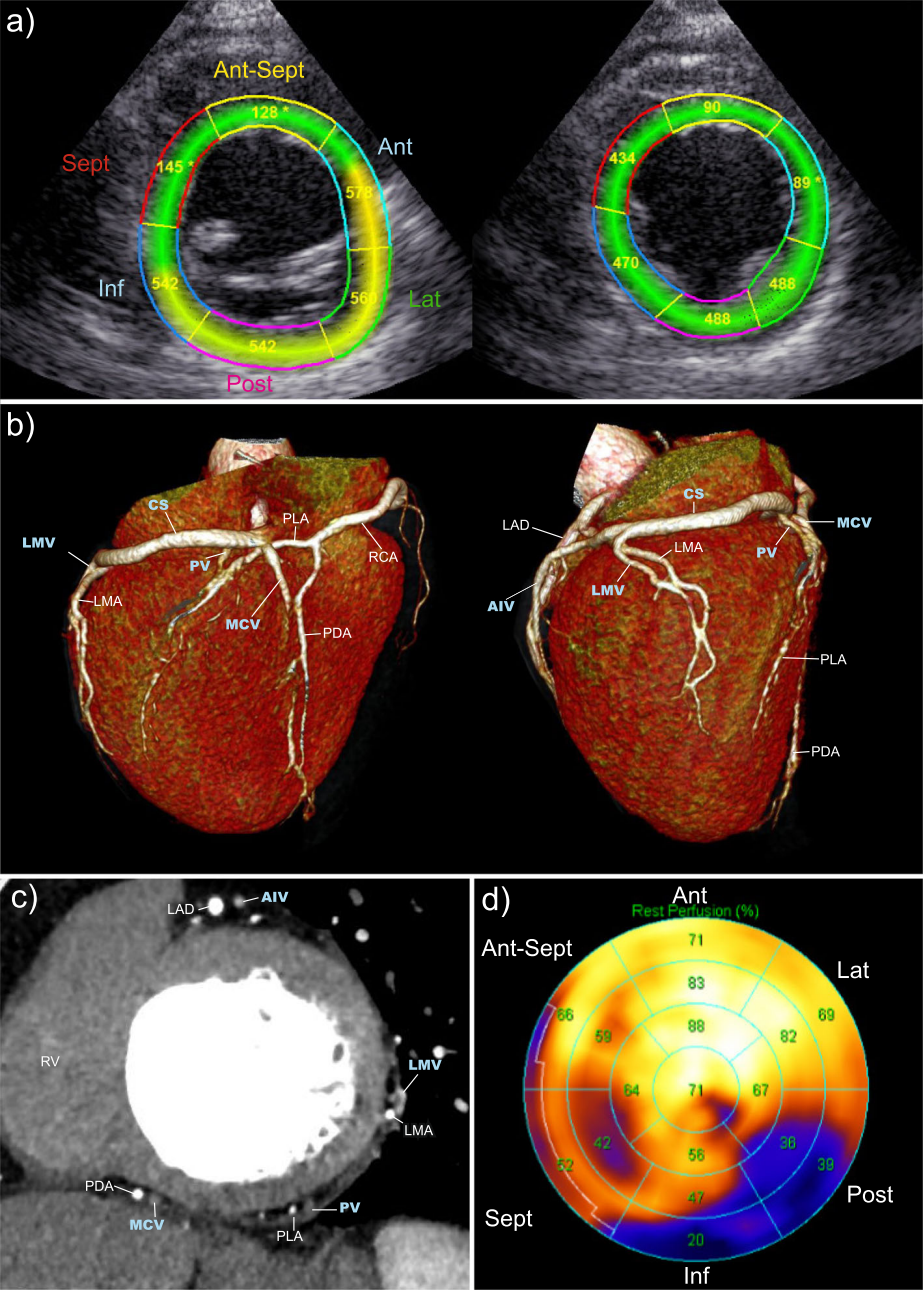
Control group: pre-implant imaging obtained to guide implantation of the left ventricular (LV) lead. **a** Echocardiographic LV short-axis view of the basal (left) and mid-LV imaging plane (right) each divided into six anatomical segments. Numbers indicate time to peak radial strain in milliseconds (ms). **b** Baseline cardiac computed tomography (CT) images in three-dimensional 3D reconstruction illustrating cardiac venous anatomy. Posterior (left) and lateral (right) view of LV. **c** Baseline cardiac CT image in the mid-LV multiplanar reformatted short-axis view illustrating cardiac venous anatomy. **d** Rubidium positron emission tomography (Rb-PET) myocardial perfusion (MP) imaging, LV 17-segment bulls-eye plot. Numbers indicate percentage of tracer-uptake; < 50% is considered as transmural scar tissue. AIV, anterior interventricular vein; Ant, anterior; Ant-Sept, antero-septal; CS, coronary sinus; Inf, inferior; LAD, left anterior descending artery; Lat, lateral; LMA, left marginal artery (circumflex artery branch); LMV, left marginal vein; MCV, middle cardiac vein; PDA, posterior descending artery (right coronary artery branch); PLA, posterolateral artery; Post, posterior; PV, posterior vein; RCA, right coronary artery; RV, right ventricle; Sept, septal

One day prior to implantation, all patients undergo identical pre-implant clinical evaluation and imaging acquisition after providing written informed consent. Patients are followed for 6 months. The study course is illustrated in Figs. 1 and 2.

### Study population

Patients are recruited at a tertiary referral center (Department of Cardiology, Aarhus University Hospital, Denmark). Consecutive patients referred to CRT-pacemaker (CRT-P) or CRT-defibrillator (CRT-D) and meeting the inclusion and exclusion criteria are eligible for study participation (Table 1).

**Table 1:** Inclusion and exclusion criteria

Inclusion criteria	
Symptomatic heart failure (NYHA class II–IV despite OMT)	
ECG with LBBB according to the Strauss criteria [37] or indwelling single or dual chamber pacemaker and a paced QRS ≥ 180 ms	
LVEF ≤ 35%	
Age ≥ 40 years	
Written informed consent	
Exclusion criteria	
Expected lifetime < 6 months	
Expected cardiac surgery within the next 6 months	
Recent (< 3 months) myocardial infarction or CABG	
Pregnant or lactating	

*NYHA* New York Heart Association, *OMT* optimal therapy for the individual patient at the time of referral for cardiac resynchronization therapy, *ECG* electrocardiogram, *LBBB* left bundle branch block, *LVEF* left ventricular ejection fraction, *CABG* coronary artery bypass graft

### Ethical considerations

The trial will be conducted according to the principles of the Helsinki Declaration II [18] and the protocol has been written in accordance with the Standard Protocol Items: Recommendation For Interventional Trials (SPIRIT) checklist (Additional file 1). The trial has been registered at ClinicalTrials.gov (identifier NCT02346097), 12 January 2015. Patients were enrolled between 16 February 2015 and 13 December 2017. The study protocol has been approved by The Central Denmark Region Committees on Health Research Ethics and by the Danish Data Protection Agency. Both implantation strategies have previously been used in clinical settings and are not known to be associated with excess risks.

### Baseline functional and clinical evaluation

Functional capacity and quality of life (QoL) are assessed by New York Heart Association (NYHA) classification [19], six-minute walk test (6MWT) [20], and Minnesota Living with Heart Failure Questionnaire (MLHFQ) using the official Danish version [21, 22].

### Echocardiography

Echocardiography is performed at baseline and 6-month follow up (6MFU) using a 3.5-MHz transducer and a commercially available system (Vivid E9, GE, Horten, Norway). Electrocardiogram (ECG)-triggered, two-dimensional (2D), tissue Doppler images (TDI), and color Doppler images are obtained during breath hold. A frame rate of 50–80 frames per second is intended. Echocardiographic images are stored in cine-loop format and analyzed offline (EchoPac BT201, GE, Horten, Norway). All measurements are averaged over three cycles. Simpson’s bi-plane method is applied to assess LV volumes and LVEF [23].

**Fig. 3:**
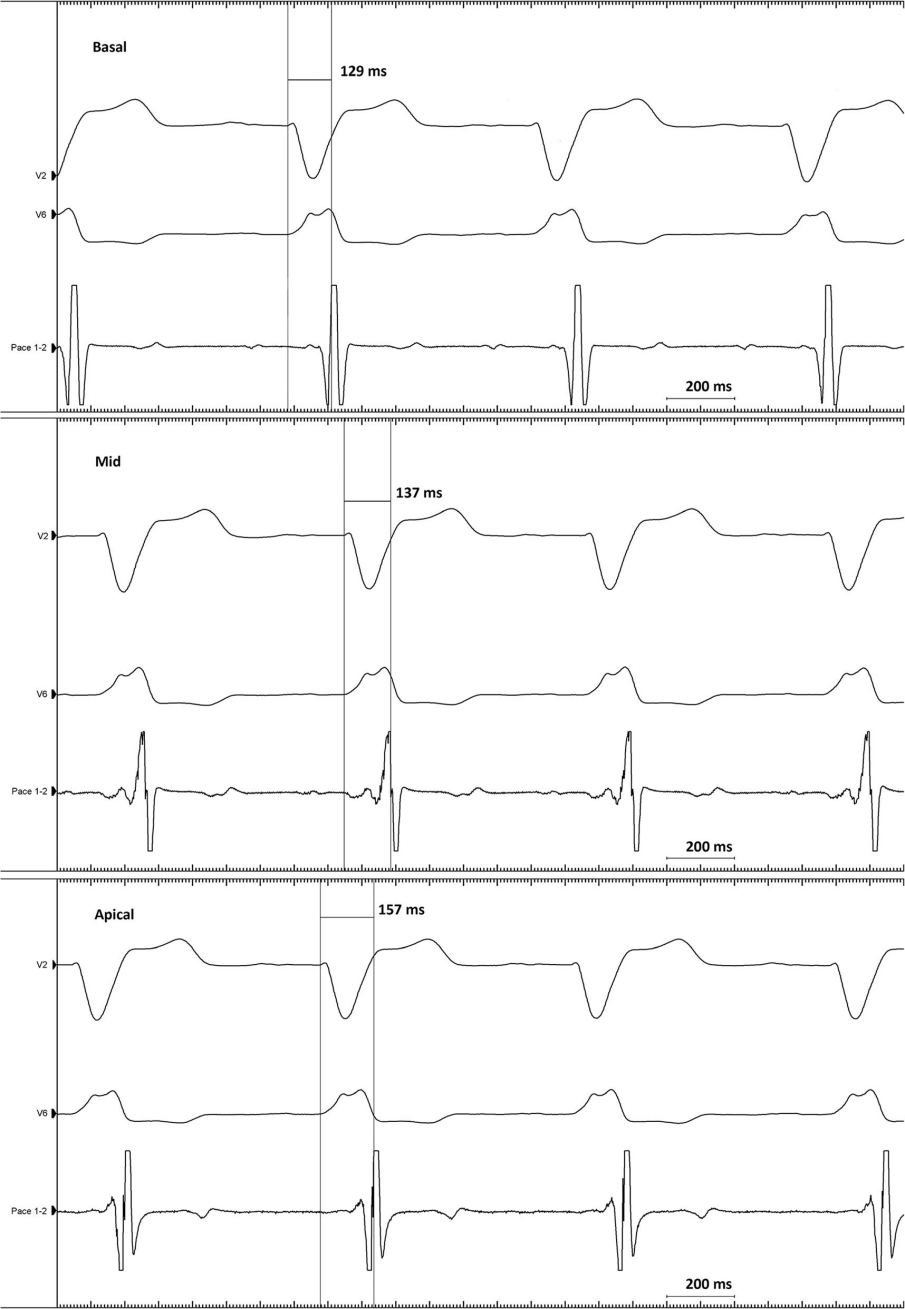
Intervention group: electrical mapping. The local electrical activation delay is measured as time in milliseconds (ms) from QRS onset in the surface 12-lead electrocardiogram (ECG) to the maximum voltage change over time recorded in the local LV electrogram (EGM), reflecting the near-field activation of the myocardium according to the LV lead (QLV interval). The figure shows three examples of QLV measurements with the LV lead in a basal, mid, and apical position, exhibiting the longest QLV interval in the apical position. For simplicity, only the surface leads V2 and V6 and the local LV EGM (Pace 1–2) are shown

The latest mechanically activated myocardial segment is determined by speckle-tracking radial strain analysis in the LV short-axis view in the basal and mid-LV imaging plane. According to the standardized LV segmentation [24], the LV short axis (SAX) is divided into six myocardial segments. Time from QRS onset to peak radial strain is measured in the basal and mid-LV segments (Fig. 3a). LV mechanical dyssynchrony is determined as the time delay between peak radial strain in the mid-LV antero-septal and the posterior segment [17]. Where radial strain analysis is not applicable in the LV SAX view (TDI), time-to-peak longitudinal strain analysis performed in the mid-LV segments in the A4Ch, A2ch and long-axis views are applied to identify the latest activated myocardial segment [25]. Echocardiograms will be analyzed by two observers, and interobserver and intraobserver variability will be reported.

### Assessment of cardiac venous anatomy and LV lead position by cardiac CT

A contrast enhanced (Optiray 350 mg/ml, Covidien, Ireland) high-pitch spiral CT scan (Siemens SOMATOM Definition Flash or Siemens SOMATOM Force, Siemens, Forchheim, Germany) is performed for pre-implant assessment of cardiac venous anatomy. Sublingual nitroglycerin is administered prior to the scan. Data are acquired with 80–140 kV tube voltage. The scans are performed ECG-gated with full pulsing only in diastole (65–80% of the RR interval), and during breath-hold. The time-tracking technique is applied to determine contrast filling in the LV cavity (20 ml for the test bolus and 50 ml for the scan). Two successive high-pitch spiral scans are performed at 15 and 19 s after peak contrast filling of the LV cavity.

The scan with the best CS visualization is used for study purposes. Images are reconstructed iteratively (Siemens SOMATOM Force) or with filtered back-projection (Siemens SOMATOM Flash). Image analysis is performed in Syngo.via, using the InSpace application (Siemens, Forchheim, Germany) for evaluation of multi-planar and three-dimensional (3D) images, respectively (Fig. 3b and c). Contrast enhanced (40 ml) high-pitch ECG-gated CT using the same scan protocol is performed the day after implantation to determine final LV lead position. Cardiac CT will not be performed in the minority of patients with depressed renal function with estimated glomerular filtration rate (eGFR) < 35 ml/min/1.73 m2., thyrotoxicosis or in the case of former severe reactions to the contrast medium.

### Localization of the myocardial scar

Pre-implant ECG-gated Rb-PET is performed in all patients using a commercially available scanner (GE Healthcare, Buckinghamshire, UK). Briefly, intravenous injection of 1150 MBq rubidium is directly followed by a 7-min list mode scan of the heart. List mode data are subsequently re-binned into 27 dynamic frames (12 × 5, 6 × 10, 4 × 20, 4 × 40, and 1 × 60 s) and one static frame (2.5–7 min following initiation of the scan). Resting MP values are calculated from dynamic Rb PET/CT images and normalized for differences in rate pressure product (RPP) using an assumed population average value of 10,000 divided by the RPP. Data are analyzed using the commercially available automatic program Quantitative PET (Cedar-Sinai Medical Center, Los Angeles, CA, USA). Static data are displayed in polar map format and analyzed using the 17-segment model (Fig. 3d) [24]. Segments with a tracer uptake < 50% are considered as transmural scar tissue, those between 50 and 75% as non-transmural scar tissue, and those ≥ 75% are considered viable myocardium [26].

### Randomization and blinding procedures

After baseline clinical evaluation and pre-implant imaging, patients are allocated either to the intervention or the control group. Study data are recorded in a web-based case record form (CRF) with logging of all data entries. The CRF is also used for randomization using computerized permuted blocks of different sizes. Randomization is stratified according to ischemic or non-ischemic heart failure etiology. An external data manager is responsible for the CRF and has programmed a computerized random-number generator.

Patients and physicians responsible for enrollment, baseline and follow-up clinical evaluation, including imaging acquisition, are blinded to the allocated treatment. Information about randomization is available only to the physician performing the implant. Data related to the device implantation, post-implant device-tests, and optimization are collected and entered into the CRF by the physicians performing the implantation and the dedicated research nurses, to ensure blinding of the investigator collecting clinical and imaging data. Information on the randomization code will be available when all patients have completed the 6MFU and all statistical analyses have been performed.

### The applied strategies for cardiac resynchronization therapy

**Fig. 4:**
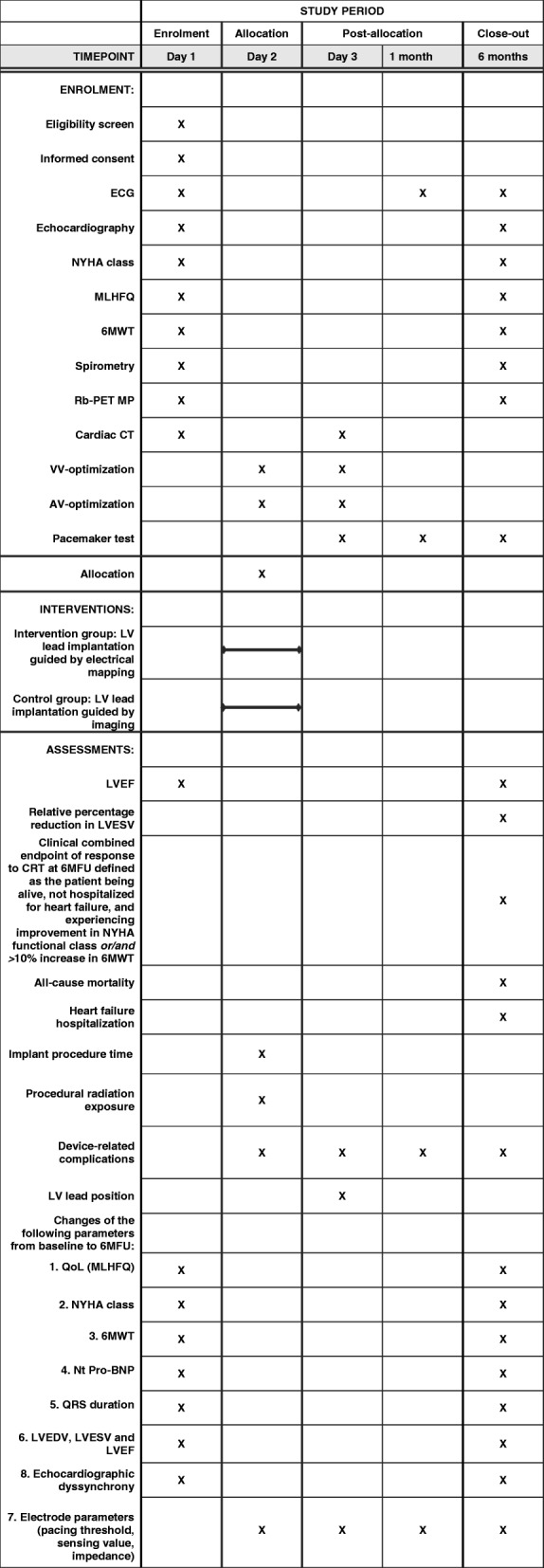
SPIRIT figure of the ElectroCRT study protocol. ECG, electrocardiogram; NYHA, New York Heart Association; MLHFQ, Minnesota Living with Heart Failure Questionnaire; 6MWT, 6-minute walk test; Rb-PET MP, rubidium positron emission tomography myocardial perfusion imaging; CT, computed tomography; VV, interventricular; AV, atrioventricular; LV, left ventricular; LVEF, left ventricular ejection fraction; LVESV, left ventricular end-systolic volume, CRT, cardiac resynchronization therapy; 6MFU, 6-month follow up; QoL, quality of life; Nt Pro-BNP, N-terminal prohormone of brain natriuretic peptide, LVEDV, left ventricular end-diastolic volume

Intervention group: prior to implantation fluoroscopic venography is co-registered to the CT venography to ensure visualization of all available CS branches. During the implantation procedure, systematic electrical activation mapping of all available epicardial veins is performed using the LV electrode and/or a specialized mapping guidewire (Visionwire®, Biotronik, Berlin, Germany). Simultaneous local LV electrograms (EGM) and 12-lead surface ECGs are acquired (CardioLab IT, GE Healthcare). The local electrical activation delay is measured as the time interval from QRS onset in the surface ECG to the maximum voltage change over time recorded in the EGM, reflecting the near-field activation of the myocardium according to the LV lead (QLV interval) [12] (Fig. 4). The QLV interval is measured in the basal, mid and apical region of each CS branch. A quadripolar LV electrode will be used as first choice in all patients. The LV electrode is implanted at the site exhibiting the longest QLV interval (and thereby the latest local electrical activation) with acceptable pacing threshold and no diaphragmatic stimulation.

Immediately after the implantation, the VVd settings are programmed by the physician performing the implantation, to obtain the narrowest QRS width. This is performed by measuring QRS width as the maximal QRS duration in any lead in the 12-lead ECG recorded at five different VVd settings: (1) right ventricle (RV) stimulation 20 ms prior to LV stimulation, (2) simultaneous biventricular pacing, (3) LV stimulation 20 ms prior to RV stimulation, (4) LV stimulation 40 ms prior to RV stimulation, and (5) LV stimulation 60 ms prior to RV stimulation.

Control group: prior to implantation the fluoroscopic venography is co-registered to the CT venography to visualize available CS branches. After combining information from echocardiography, Rb-PET, and cardiac CT, the LV electrode is targeted towards the CS branch closest to the latest mechanically activated non-scarred myocardial segment. Basal and mid-LV segments of the CS tributaries are prioritized separately according to the relation with basal and mid-LV segmental mechanical activation delay. CT images of cardiac venous anatomy are available to the physician performing the implantation in a 2D short-axis view and in 3D volume-rendered reconstructions. The CS tributary closest to the optimal LV pacing site is labeled as first priority for LV lead placement. Tributaries closest to the second, third, or fourth latest activated segment with viable myocardium are chosen as the second, third, or fourth priority [17].

In the imaging group, the VVd is programmed to simultaneous biventricular stimulation. In both treatment groups, atrioventricular (AV) optimization is performed the day after implantation using the iterative method ensuring maximal separation of the E-wave and A-wave without termination of the A-wave [27].

### Endpoints

The primary endpoint is absolute change in left ventricular ejection fraction (LVEF) from baseline to 6MFU measured by 2D echocardiography. Secondary outcomes include relative percentage reduction in LV end-systolic volume (LVESV) and a combined clinical outcome measure of response to CRT at 6MFU defined as the patient being alive, not hospitalized for heart failure, and experiencing an improvement in NYHA functional class or/and > 10% increase in 6MWT [17]. Hospitalization for heart failure is defined as admission to hospital for more than 24 h with symptoms of congestive heart failure and need for intensified drug treatment for heart failure. Other secondary outcome measures include:

- All-cause mortality.
- Hospitalization for heart failure.
- Implantation procedure time.
- Procedural radiation exposure.
- Device-related complications.
- Indices of dyssynchrony and association between dyssynchrony, LV lead placement, and response to CRT.
- Changes in QoL, NYHA functional class, 6MWT, N-terminal prohormone of brain natriuretic peptide (Nt Pro-BNP), QRS duration, LV end-diastolic volume (LVEDV), LVESV, LVEF, mechanical dyssynchrony, and LV lead parameters (pacing threshold, sensing value, impedance).

### Sample size

We hypothesize that the intervention group strategy will result in an excess increase in LVEF of 4% compared with the strategy used in the control group, where an 8% increase in LVEF is expected [9]. To identify this absolute increase in LVEF of 12% in the intervention group and to achieve statistical power of 80%, the study will need a sample size of 98 patients, given a standard deviation (SD) of 7% in both groups, and a two-sided alpha value of 0.05. To achieve statistical power of 80% for the secondary endpoint of relative reduction in LVESV a sample size of 116 patients is needed, when assuming a 33% reduction in the control group and a 45% reduction in the intervention group (expected SD of 23% in both groups and given a two-sided alpha value of 0.05%). To achieve statistical power of > 80% with a margin of non-inferiority of 20% for the secondary endpoint of clinical response to CRT (assuming a 75% clinical response rate in the control group) we will need a sample size of 116 patients (given a two-sided alpha value of 0.05%). Taking into consideration expected loss to follow up in approximately 5% of patients, 122 patients are included.

### Statistical analysis

All analyses will be conducted according to the intention-to-treat principle and will be performed before breaking the randomization code. Baseline characteristics are presented and compared clinically. Primary and secondary endpoints will be analyzed using linear regression for continuous variables and logistic regression for binary outcome measures, including heart failure etiology as a variable in the model. Multivariate regression analysis will be reported for absolute change in LVEF including baseline LVEF, heart failure etiology, sex, and baseline QRS width as covariates. Predictors of missing outcome values will be identified by comparing baseline characteristics and will be included in a multivariate regression model for sensitivity analysis. Intraclass correlation coefficients are computed to assess intraobserver and interobserver agreement for the echocardiographic parameters included in the primary endpoint: LVEF, LVEDV, LVESV assessed for 20 echocardiograms. A two-sided *P* value < 0.05 is considered significant.

## Discussion

### Electrically guided CRT strategy

Positioning of the LV lead according to the latest activated myocardial segment has been shown to be an important determinant of response to CRT. In this current study, the strategy is to target the myocardial region with the latest electrical activation. The advantage of this approach is that the electrical data obtained from mapping are readily available during the implant procedure without the need for alignment of imaging modalities to target a specific myocardial segment. Several observational studies indicate that a LV lead location in regions with late electrical activation is associated with improved clinical outcome and reverse remodeling [12, 13, 28]. No randomized studies have evaluated the impact of systematic electrical mapping of available CS branches to target the segment with the latest electrical activation. In addition, post-implant device programming in the VVd producing the shortest biventricular paced QRS duration will be applied to optimize the electrical resynchronization suggested to increase the response to CRT [16]. The impact of this combined electrical CRT optimization strategy has not previously been evaluated.

### Imaging-guided LV lead placement

Randomized studies have demonstrated that targeting LV lead placement towards the latest mechanically activated non-scarred myocardial area as assessed by speckle-tracking echocardiography strain analysis improves clinical outcome and LV reverse remodeling compared with a routine anatomical approach targeting the non-apical posterolateral region [9, 11]. Similar findings have been observed using a multimodality imaging-guided approach combining speckle-tracking echocardiography, MP imaging, and cardiac CT to target the cardiac vein closest to the latest contracting non-scarred myocardial region [10, 17]. However, echocardiographic measurement of the myocardial mechanical activation pattern may be difficult to align with fluoroscopic imaging during the implant procedure [29] and may have substantial degree of interobserver variability [30]. Furthermore, pre-implant imaging is costly and time consuming.

Due to the superior outcome associated with the imaging-guided approach as compared with conventional LV lead placement [9–11], we applied the imaging-guided approach as our control group strategy. This was done to evaluate the potential benefit of the simpler electrically guided CRT optimization strategy not limited by the need for pre-implant imaging and alignment of imaging modalities.

### Methods used in the ElectroCRT study

The extent of myocardial scar tissue is inversely related to the response to CRT [31] and placement of the LV lead in a myocardial region with scar tissue is associated with poor CRT outcome [32]. Rb-PET is well-established for visualizing regions [26, 33] and distribution of myocardial scar [34], but it has lower spatial resolution than cardiac magnetic resonance (MR). However, we chose to assess scar with MP imaging, as a substantial proportion of our patients are not eligible for cardiac MR due to an indwelling device. Myocardial scar as detected by MP imaging is not considered in the electrically guided intervention group when implanting the LV lead, to ensure complete separation of the electrically and imaging-guided strategy.

Pre-implant cardiac CT is applied to assess CS anatomy as potentially available CS tributaries may be missed during fluoroscopic balloon occlusive angiography and to ease planning implantation of the LV lead. Post-implant cardiac CT is performed to determine the final LV pacing site because fluoroscopy has been shown to be inaccurate and poorly reproducible for this purpose [35].

### Choice of endpoint

Considering the complex design, this trial would be difficult to conduct as a multi-center study, and therefore it does not have statistical power to investigate differences between groups in “harder” endpoints such as survival or hospitalization due to heart failure. We chose LVEF as the primary endpoint, because it is a commonly used and universally understood echocardiographic measure of LV systolic function, which furthermore is a good predictor of survival in patients with CRT [36]. Accordingly, the findings in this study may generate hypotheses to be tested in future larger-scale, multi-center, randomized studies.

### Limitations of the ElectroCRT study

We acknowledge the inherent limitations of a single-center study design. Nevertheless, the double-blinded, randomized, controlled trial design for comparing two advanced strategies for CRT optimization is the strongest research instrument to investigate a difference in outcome caused by these interventions.

We do not merge the applied imaging modalities and the procedural fluoroscopy in the control group. However, we use the standard myocardial segmentation [24] to ease an approximated alignment of the echocardiography, MP imaging, 2D and 3D cardiac CT reconstructions of cardiac venous anatomy and procedural fluoroscopy [10].

The introduction of cardiac CT and Rb-PET prior to CRT will increase the patient’s cumulative radiation exposure. Several approaches to minimize radiation dose are applied in this study, including the use of iterative reconstruction algorithms, application of prospectively triggered high-pitch CT scans, individual settings of tube voltage and current, and the use of Rb-PET for MP imaging, respectively. Furthermore, the utility and potential benefits of participating in this study are expected to equalize the risks of exposure to ionizing radiation, possible adverse effects, and inconvenience to the patients.

### Perspective

No randomized trial has investigated the effect of a CRT implant-strategy targeting optimal electrical resynchronization achieved by guiding LV lead placement to the myocardial region with the latest electrical activation combined with post-implant VVd optimization for narrowing the paced QRS width. If an excess increase in LVEF is achieved by LV lead implantation guided by systematic electrical mapping followed by VVd optimization for narrowing paced QRS, this study supports the conduct of larger, multicenter, randomized clinical trials investigating the impact of electrically guided LV lead implantation on clinical outcomes in patients treated with CRT.

## Trial status

Patients were enrolled between 16 February 2015 and 13 December 2017. The current protocol article was submitted to *Trials* before all patients were enrolled and before analyzing any data and breaking the randomization code.

## Additional file

Additional file 1: SPIRIT checklist of the ElectroCRT study protocol. (PDF 174 kb)

## Abbreviations

2D: Two-dimensional
3D: Three-dimensional
6MFU: Six-month follow-up
6MWT: Six-minute walk test
AIV: Anterior interventricular vein
Ant: Anterior
Ant-Sept: Antero-septal
AV: Atrioventricular
CABG: Coronary artery bypass Graft
CRF: Case record form
CRT: Cardiac resynchronization therapy
CRT-D: Cardiac resynchronization therapy defibrillator
CRT-P: Cardiac resynchronization therapy pacemaker
CS: Coronary sinus
CT: Computed tomography
ECG: Electrocardiogram
eGFR: Estimated glomerular filtration Rate
EGM: Electrogram
Inf: Inferior
LAD: Left anterior descending artery
Lat: Lateral
LBBB: Left bundle branch block
LMA: Left marginal artery (circumflex artery branch)
LMV: Left marginal vein
LVEDV: Left ventricular end-diastolic volume
LVEF: Left ventricular ejection fraction
LVESV: Left ventricular end-systolic volume
MBq: Mega Becquerel
MCV: Middle cardiac vein
mHz: Megahertz
ml: Milliliters
MLHFQ: Minnesota Living with Heart Failure Questionnaire
MP: Myocardial perfusion
MR: Magnetic resonance
ms: Milliseconds
Nt Pro-BNP: N-terminal prohormone of brain natriuretic peptide
NYHA: New York Heart Association
OMT: Optimal medical treatment
PDA: Posterior descending artery (right coronary artery branch)
PLA: Posterolateral artery
Post: Posterior
PV: Posterior vein
QLV interval: The interval from onset of the QRS on the surface 12-lead ECG to the maximum voltage change over time recorded in the EGM, reflecting the near-field activation of the myocardium according to the LV lead
QoL: Quality of life
Rb-PET: ^82^Rubidium positron emission tomography
RCA: Right coronary artery
RV: Right ventricle/right ventricular
SAX: Short axis view
Sept: Septal
SPIRIT: Standard Protocol Items: Recommendation For Interventional Trials
TDI: Tissue Doppler Images
VVd: Interventricular pacing delay

## References

[1] Abraham WT, Fisher WG, Smith AL, Delurgio DB, Leon AR, Loh E (2002). Cardiac resynchronization in chronic heart failure. N Engl J Med.

[2] Bristow MR, Saxon LA, Boehmer J, Krueger S, Kass DA, De Marco T (2004). Cardiac-resynchronization therapy with or without an implantable defibrillator in advanced chronic heart failure. N Engl J Med.

[3] Cleland JG, Daubert JC, Erdmann E, Freemantle N, Gras D, Kappenberger L (2005). The effect of cardiac resynchronization on morbidity and mortality in heart failure. N Engl J Med.

[4] Brignole M, Auricchio A, Baron-Esquivias G, Bordachar P, European Society of Cardiology (ESC), European Heart Rhythm Association (EHRA) (2013). ESC guidelines on cardiac pacing and cardiac resynchronization therapy: the task force on cardiac pacing and resynchronization therapy of the European Society of Cardiology (ESC). Developed in collaboration with the European Heart Rhythm Association (EHRA). Europace.

[5] Kirkfeldt R. E., Johansen J. B., Nohr E. A., Jorgensen O. D., Nielsen J. C. (2013). Complications after cardiac implantable electronic device implantations: an analysis of a complete, nationwide cohort in Denmark. European Heart Journal.

[6] Kronborg MB, Mortensen PT, Kirkfeldt RE, Nielsen JC (2008). Very long term follow-up of cardiac resynchronization therapy: clinical outcome and predictors of mortality. Eur J Heart Fail.

[7] Yu CM, Sanderson JE, Gorcsan J (2010). Echocardiography, dyssynchrony, and the response to cardiac resynchronization therapy. Eur Heart J.

[8] Mullens W, Grimm RA, Verga T, Dresing T, Starling RC, Wilkoff BL (2009). Insights from a cardiac resynchronization optimization clinic as part of a heart failure disease management program. J Am Coll Cardiol.

[9] Khan FZ, Virdee MS, Palmer CR, Pugh PJ, O'Halloran D, Elsik M (2012). Targeted left ventricular lead placement to guide cardiac resynchronization therapy: the TARGET study: a randomized, controlled trial. J Am Coll Cardiol.

[10] Sommer Anders, Kronborg Mads Brix, Nørgaard Bjarne Linde, Poulsen Steen Hvitfeldt, Bouchelouche Kirsten, Böttcher Morten, Jensen Henrik Kjaerulf, Jensen Jesper Møller, Kristensen Jens, Gerdes Christian, Mortensen Peter Thomas, Nielsen Jens Cosedis (2016). Multimodality imaging-guided left ventricular lead placement in cardiac resynchronization therapy: a randomized controlled trial. European Journal of Heart Failure.

[11] Saba S, Marek J, Schwartzman D, Jain S, Adelstein E, White P (2013). Echocardiography-guided left ventricular lead placement for cardiac resynchronization therapy: results of the Speckle Tracking Assisted Resynchronization Therapy for Electrode Region trial. Circ Heart Fail.

[12] Gold MR, Birgersdotter-Green U, Singh JP, Ellenbogen KA, Yu Y, Meyer TE (2011). The relationship between ventricular electrical delay and left ventricular remodelling with cardiac resynchronization therapy. Eur Heart J.

[13] Kandala J, Upadhyay GA, Altman RK, Parks KA, Orencole M, Mela T (2013). QRS morphology, left ventricular lead location, and clinical outcome in patients receiving cardiac resynchronization therapy. Eur Heart J.

[14] Kronborg MB, Nielsen JC, Mortensen PT (2010). Electrocardiographic patterns and long-term clinical outcome in cardiac resynchronization therapy. Europace.

[15] Tamborero D, Vidal B, Tolosana JM, Sitges M, Berruezo A, Silva E (2011). Electrocardiographic versus echocardiographic optimization of the interventricular pacing delay in patients undergoing cardiac resynchronization therapy. J Cardiovasc Electrophysiol.

[16] Tamborero D, Mont L, Sitges M, Silva E, Berruezo A, Vidal B (2009). Optimization of the interventricular delay in cardiac resynchronization therapy using the QRS width. Am J Cardiol.

[17] Sommer A, Kronborg MB, Poulsen SH, Bottcher M, Norgaard BL, Bouchelouche K (2013). Empiric versus imaging guided left ventricular lead placement in cardiac resynchronization therapy (ImagingCRT): study protocol for a randomized controlled trial. Trials.

[18] WMA: Declaration of Helsinki - ethical principles for medical research involving human subjects. https://www.wma.net/policies-post/wma-declaration-of-helsinki-ethical-principles-for-medical-research-involving-human-subjects/. 19886379

[19] The Criteria Committee of the New York Heart Association (1964). Nomenclature and criteria for diagnosis and diseases of the heart and great vessels.

[20] ATS Committee on Proficiency Standards for Clinical Pulmonary Function Laboratories (2002). ATS statement: guidelines for the six-minute walk test. Am J Respir Crit Care Med.

[21] Rector Thomas S., Tschumperlin Linda K., Kubo Spencer H., Bank Alan J., Francis Gary S., McDonald Kenneth M., Keeler Carol A., Silver Marc A. (1995). Use of the living with heart failure questionnaire to ascertain patients' perspectives on improvement in quality of life versus risk of drug-induced death. Journal of Cardiac Failure.

[22] Rector TS, Anand IS, Cohn JN (2006). Relationships between clinical assessments and patients’ perceptions of the effects of heart failure on their quality of life. J Card Fail.

[23] Schiller NB, Shah PM, Crawford M, DeMaria A, Devereux R, Feigenbaum H (1989). Recommendations for quantitation of the left ventricle by two-dimensional echocardiography. American Society of Echocardiography Committee on Standards, Subcommittee on Quantitation of Two-Dimensional Echocardiograms. J Am Soc Echocardiogr.

[24] Cerqueira MD, Weissman NJ, Dilsizian V, Jacobs AK, Kaul S, Laskey WK (2002). Standardized myocardial segmentation and nomenclature for tomographic imaging of the heart. A statement for healthcare professionals from the Cardiac Imaging Committee of the Council on Clinical Cardiology of the American Heart Association. Circulation.

[25] Murphy RT, Sigurdsson G, Mulamalla S, Agler D, Popovic ZB, Starling RC (2006). Tissue synchronization imaging and optimal left ventricular pacing site in cardiac resynchronization therapy. Am J Cardiol.

[26] vom Dahl J, Muzik O, Wolfe ER, Allman C, Hutchins G, Schwaiger M (1996). Myocardial rubidium-82 tissue kinetics assessed by dynamic positron emission tomography as a marker of myocardial cell membrane integrity and viability. Circulation.

[27] Gorcsan J, Abraham T, Agler DA, Bax JJ, Derumeaux G, Grimm RA (2008). Echocardiography for cardiac resynchronization therapy: recommendations for performance and reporting–a report from the American Society of Echocardiography Dyssynchrony Writing Group endorsed by the Heart Rhythm Society. J Am Soc Echocardiogr.

[28] Liang Y, Yu H, Zhou W, Xu G, Sun YI, Liu R (2015). Left ventricular lead placement targeted at the latest activated site guided by electrophysiological mapping in coronary sinus branches improves response to cardiac resynchronization therapy. J Cardiovasc Electrophysiol.

[29] Behar JM, Claridge S, Jackson T, Sieniewicz B, Porter B, Webb J (2017). The role of multi modality imaging in selecting patients and guiding lead placement for the delivery of cardiac resynchronization therapy. Expert Rev Cardiovasc Ther.

[30] Chung ES, Leon AR, Tavazzi L, Sun JP, Nihoyannopoulos P, Merlino J (2008). Results of the Predictors of Response to CRT (PROSPECT) trial. Circulation.

[31] Ypenburg C, Schalij MJ, Bleeker GB, Steendijk P, Boersma E, Dibbets-Schneider P (2007). Impact of viability and scar tissue on response to cardiac resynchronization therapy in ischaemic heart failure patients. Eur Heart J.

[32] Delgado V, van Bommel RJ, Bertini M, Borleffs CJ, Marsan NA, Arnold CT (2011). Relative merits of left ventricular dyssynchrony, left ventricular lead position, and myocardial scar to predict long-term survival of ischemic heart failure patients undergoing cardiac resynchronization therapy. Circulation.

[33] Anagnostopoulos C, Georgakopoulos A, Pianou N, Nekolla SG (2013). Assessment of myocardial perfusion and viability by positron emission tomography. Int J Cardiol.

[34] Qayyum AA, Hasbak P, Larsson HB, Christensen TE, Ghotbi AA, Mathiasen AB (2014). Quantification of myocardial perfusion using cardiac magnetic resonance imaging correlates significantly to rubidium-82 positron emission tomography in patients with severe coronary artery disease: a preliminary study. Eur J Radiol.

[35] Sommer A., Kronborg M. B., Norgaard B. L., Gerdes C., Mortensen P. T., Nielsen J. C. (2014). Left and right ventricular lead positions are imprecisely determined by fluoroscopy in cardiac resynchronization therapy: a comparison with cardiac computed tomography. Europace.

[36] Solomon SD, Foster E, Bourgoun M, Shah A, Viloria E, Brown MW (2010). Effect of cardiac resynchronization therapy on reverse remodeling and relation to outcome: multicenter automatic defibrillator implantation trial: cardiac resynchronization therapy. Circulation.

[37] Strauss DG, Selvester RH, Wagner GS (2011). Defining left bundle branch block in the era of cardiac resynchronization therapy. Am J Cardiol.

